# Biomarkers of Checkpoint Inhibitor Induced Immune-Related Adverse Events—A Comprehensive Review

**DOI:** 10.3389/fonc.2020.585311

**Published:** 2021-02-11

**Authors:** Josefien W. Hommes, Rik J. Verheijden, Karijn P. M. Suijkerbuijk, Dörte Hamann

**Affiliations:** ^1^ Center for Translational Immunology, University Medical Center Utrecht, Utrecht, Netherlands; ^2^ Department of Medical Oncology, Cancer Center, University Medical Center Utrecht, Utrecht, Netherlands; ^3^ Central Diagnostic Laboratory, University Medical Center Utrecht, Utrecht, Netherlands

**Keywords:** biomarker, immune checkpoint inhibition, immunotherapy, cytokines, blood cells, review (article), checkpoint inhibitor toxicity, immune-related adverse event

## Abstract

Immune checkpoint inhibitors (ICIs) have substantially improved the prognosis of patients with different types of cancer. Through blockade of cytotoxic T-lymphocyte antigen 4 (CTLA-4) and programmed cell death protein 1 (PD-1), negative feedback mechanisms of the immune system are inhibited, potentially resulting in very durable anti-tumor responses. Despite their promise, ICIs can also elicit auto-immune toxicities. These immune-related adverse events (irAEs) can be severe and sometimes even fatal. Therefore, being able to predict severe irAEs in patients would be of added value in clinical decision making. A search was performed using “adverse events”, “immune checkpoint inhibitor”, “biomarker”, and synonyms in PubMed, yielding 3580 search results. After screening title and abstract on the relevance to the review question, statistical significance of reported potential biomarkers, and evaluation of the remaining full papers, 35 articles were included. Five additional reports were obtained by means of citations and by using the similar article function on PubMed. The current knowledge is presented in comprehensive tables summarizing blood-based, immunogenetic and microbial biomarkers predicting irAEs prior to and during ICI therapy. Until now, no single biomarker has proven to be sufficiently predictive for irAE development. Recommendations for further research on this topic are presented.

## Introduction

Since the U.S. Food and Drug administration (FDA)’s approval of ipilimumab for metastatic melanoma patients in 2011, immune checkpoint inhibitors (ICIs) have become an important treatment for many cancer patients (1). Since then, ICIs have been approved for a wide range of cancer types, including melanoma, kidney cancer, lung cancer, lymphoma, and liver cancer (1). Immune checkpoints successfully targeted by ICIs are cytotoxic T lymphocyte antigen 4 (CTLA-4) and programmed cell death protein 1 (PD-1).

Immune checkpoints play an important role in immune homeostasis by controlling immune responses, maintaining self-tolerance and preventing autoimmunity. ICIs are monoclonal antibodies that specifically target immune checkpoints and block their function. Upon initial response of a T cell to an antigen, CTLA-4 is upregulated on its membrane and competes with CD28 for binding B7-1 (CD80) and B7-2 (CD86) on antigen presenting cells (APCs) by binding with higher affinity (2). In contrast to CD28 which is a costimulatory factor on T cells, CTLA-4 inhibits further activation of effector T cells. Furthermore, CTLA-4 expression on regulatory T cells (Tregs) may result in trans-endocytosis of B7-1 and B7-2 on APCs, thereby leaving APCs without costimulatory factors. PD-1, upon binding its ligands (PD-L1 and to a lesser extend PD-L2), which are mainly present on non-lymphoid cells in peripheral tissues, generates local tolerance by dephosphorylating the T-cell receptor, leading to T-cell exhaustion (2). By blocking the above described tolerance mechanisms, ICIs enforce anti-tumor immunity, which has clinically proven to result in long-lasting responses even after stopping treatment.

As a consequence of their mechanism of action, ICIs can cause immune-related adverse events (irAEs). The onset of irAEs is highly unpredictable, as they may develop early after ICI treatment up to more than 18 months after treatment started (3, 4). Furthermore, patients do not necessarily develop a single irAE, but may develop multiple different irAEs, either simultaneously or subsequently (5). Severity of irAEs is graded according to the Common Terminology Criteria for Adverse Events (CTCAE) on a scale from 1 (mild) to 5 (death) (6). IrAE frequency differs per ICI treatment, with any irAE occurring in 60%–85% of anti-CTLA-4 treated patients, 57%–85% of anti-PD-1 treated patients, and 95% in patients receiving combined CTLA-4 and PD-1 blockade (7). Severe irAEs (≥grade 3) occur in approximately 10%–27% of anti-CTLA-4 treated patients, 7%–20% of anti-PD-1 treated patients, and 55% of patients receiving combined anti-CTLA-4 and anti-PD-1 (7). Patterns of irAEs differ per ICI type. As an example, colitis occurs more frequently in anti-CTLA-4 treated patients while thyroid disorders are more frequently seen during anti-PD-1 therapy (8). While CTLA-4 is thought to inhibit immune responses in an earlier phase, PD-1 inhibits T cells at a later stage in the peripheral tissue. Although it has not been fully understood why irAE profiles differ between anti-CTLA-4 and anti-PD-1 treated patients, some hypotheses have been proposed (4). As an example, the higher frequency of autoantibody-related irAEs such as thyroid disorders in PD-1 treated patients could be a result of the modulation of humoral immunity by PD-1-inhibitors, or of its effects on self-tolerance. Keeping this in mind, biomarkers could very well be ICI type-specific. This hypothesis is supported by data showing that anti-CTLA-4-induced and anti-PD-1-induced colitis are eminently different in their immune cell composition, suggesting a distinct underlying mechanism for these toxicities (9).

Frequently observed irAEs include dermatitis, colitis, and thyroiditis, while especially the more rare irAEs, such as myocarditis, myositis, and encephalitis have a high fatality rate (5). It has been suggested that irAE kinetics differ per organ type. Dermatological irAEs usually develop early, followed by gastrointestinal irAEs such as colitis (after 1 to 3 months), with hepatitis and endocrinopathies occurring later (10). Usually, irAEs are diagnosed once patients experience symptoms, and after alternative diagnoses are ruled out by additional testing, such as imaging, endoscopic evaluation, biopsies or blood tests (11, 12). Treatment depends on the severity of the irAE, and can include temporary or permanent ICI discontinuation, corticosteroids and second line immunosuppressants (3).

With more ICIs being approved and increasing indications, patients and healthcare professionals will progressively be confronted with irAEs. However, diagnosing irAEs is challenging due to their highly variable and often aspecific clinical presentation, which complicates distinguishing irAEs from alternative diagnoses such as infection or tumor progression. This often leads to delay in diagnosis and since early immunosuppressive treatments for irAEs can prevent morbidity and even mortality, biomarkers that can predict or signal irAEs in an early stage are urgently needed (13). A biomarker is defined as a characteristic measured as an indicator of pathogenic processes or responses to an exposure or intervention (14). While starting with proof that a biomarker is statistically associated with the clinical state of interest (i.e., irAEs), subsequent assessment of its diagnostic or predictive accuracy is essential to determine clinical utility. Two types of biomarkers can be distinguished: biomarkers assessed either at baseline or during treatment that predict irAEs, or biomarkers that can be used to signal/diagnose irAEs at the moment of signs or symptoms during treatment. Biomarkers determining the risk of irAE development prior to the start of therapy could be used to stratify patients offering alternative treatments, or monotherapy instead of combination ICIs to patients who are predicted to be at high-risk. However, sufficient discriminative power is a prerequisite for its use in clinical decision making. If, for example, a two times increased risk of severe irAEs is predicted, alternative treatment strategies could be considered (including refraining from ICIs in the adjuvant setting) as well as more intensive clinical monitoring of the patients by more frequent and lower ICI dosing. A biomarker during treatment, signaling upcoming toxicity, could serve as a warning upon which patients could be monitored more strictly, ICIs could be discontinued early or immunosuppressive therapy could be started more rapidly. However, a particularly strong correlation with both timing and severity of toxicity is required to take such far-reaching decisions. Finally, a biomarker that can help to adequately diagnose irAEs at the moment of signs or symptoms during ICI treatment could prevent delay in diagnosis and enable early immunosuppressive management.

In the last years, several studies have been performed searching for potential irAE biomarkers. Recently, two reviews on irAE biomarkers have been published (15, 16). However, these reviews did not include all studies reported. Particularly, studies describing biomarkers during ICI treatment were lacking. In this review we aim to provide an overview of primary articles on blood-based and microbial biomarkers described so far. We performed a search using “adverse events”, “immune checkpoint inhibitor”, “biomarker”, and synonyms in PubMed, yielding 3580 search results (see Appendix 1 for complete search). After screening title and abstract on the relevance to the review question and evaluation of the remaining full papers, 35 articles were included. Five additional reports were obtained by means of citations and by using the similar article function on PubMed. We excluded case reports and included only papers with statistically substantiated data. The majority of studies analyzed in this review, focused on predictive biomarkers.

## Biomarkers

An overview of the studies on irAE biomarkers at baseline and during treatment is given in **Tables 1** and **2**, respectively. The studies are discussed in more detail below.

**Table 1:** Potential biomarkers for irAE development measured prior to ICI treatment.

Potential biomarker	Determinant	Results	Significance	Accuracy	Cancer type	n	Treatment	Study design	References
*Cellular*									
Neutrophil to lymphocyte ratio (NLR)	Ratios calculated from peripheral neutrophil, eosinophils and lymphocyte count; and albumin	NLR ≥3 associated with less irAEs	OR_adj_ = 0.37; 95%CI 0.17–0.81; p = 0.012	–	Solid tumor	391	Anti-PD-1	Retrospective cohort	(17)
NLR; prognostic nutrition index (PNI)	Ratio calculated from neutrophil and lymphocyte counts; LDH and serum albumin	Low NLR (<5) and high PNI (≥45) independently associated with irAEs	p < 0.001 | p = 0.001	–	NSCLC	102	Anti-PD-1	Retrospective cohort	(18)
Platelet to lymphocyte ratio (PLR)	Ratios calculated from peripheral neutrophil, lymphocyte, and platelet counts	PLR <180 associated with irAEs	OR_adj_ = 2.3; 95%CI 1.1–4.8; p = 0.027	–	NSCLC	173	Anti-PD-(L)1	Retrospective cohort	(19)
Eosinophils	Peripheral blood cell counts	Eosinophil count associated with grade ≥2 irAEs	OR_adj_ = 1.003; 95%CI 1.000–1.006; p = 0.027	–	Solid tumor	167	Anti-PD-1, combination	Retrospective cohort	(20)
	Peripheral blood cell counts	Eosinophil count of >240/μl associated with endocrine (but not any) irAEs	OR = 7.0; 95%CI 1.50–32.72; p = 0.0134	Sens = 88% Spec = 50%	Advanced melanoma	44	Anti-PD-1	Retrospective cohort	(21)
Lymphocytes	Peripheral blood cell counts	Lymphocyte count >2,000 associated with risk of grade ≥2 irAEs	OR_adj_ = 1.996; 95%CI 1.16–3.49; p = 0.014	–	Solid tumor	167	Anti-PD-1, combination	Retrospective cohort	(20)
	Myeloid and T cell subsets by flow cytometry	Lower % PD-1 expression on CD4+ and CD8+ T cells associated with grade ≥3 irAEs	p = 0.025 | p = 0.022	–	Metastatic melanoma	44	Anti-CTLA-4	Retrospective cohort	(22)
	CD4+ T cells and Treg cells counts at baseline	Lower percentage of Tregs associated with colitis	p = 0.018	–	Metastatic melanoma	18	Anti-CTLA-4	Prospective cohort	(23)
*Cytokines/Chemokines*									
IL-6	5 cytokines and lymphocyte subsets	Lower IL-6 levels associated with grade ≥3 irAEs	OR = 2.84; 95%CI 1.34–6.03; p = 0.007	Sens = 70% Spec = 66%	Metastatic melanoma	140	Anti-CTLA-4	Prospective cohort	(24)
IL-6, IL-8, and sCD25	Baseline IL-6, IL-8 and sCD25 in serum	Lower levels of IL-6, IL-8, and sCD25 associated with colitis	p = 0.008 | p = 0.0031 | p = 0.0097	–	Melanoma	18	Anti-CTLA-4	Prospective cohort	(23)
IL-17	Multiplex serum testing for 36 different cytokines and chemokines	Higher IL-17 levels associated with grade ≥3 colitis and grade ≥3 irAEs	p = 0.02 | p = 0.03	–	Advanced melanoma	35	Anti-CTLA-4	Clinical trial	(25)
Various cytokines/chemokines	65 cytokines/chemokines profiles; validated in separate cohort	CYTOX score (G-CSF, GM-CSF, fractalkine, FGF-2, IFNα2, IL1a, IL1B, IL1RA, IL2, IL12p70 and IL13) associated with irAEs that required ICI discontinuation or immunosuppression	p = 0.0366	AUC = 0.68	Melanoma	49	Anti-PD-1 or combination	Prospective cohort	(26)
IL-1β, IL-2, GM-CSF	13 cytokines in serum	Increased levels of IL-1β, IL-2, and GM-CSF correlated with thyroid irAEs	p < 0.05	–	Advanced malignancies	26	Anti-PD-1, anti-CTLA-4, or combination	Prospective cohort	(27)
CXCL9, CXCL10, CXCL11, CCL19	40 cytokines in serum	Lower levels of CXCL9, CXCL10, CXCL11, and CCL19 associated with irAEs	p < 0.05	–	Solid tumor	42	Anti-PD-(L)1	Prospective cohort	(28)
*Autoantibodies*									
Anti-thyroid antibodies	Thyroid peroxidase and thyroglobulin antibodies in serum	Anti-Tg and anti-TPO associated with thyroid dysfunction	p < 0.001 | p = 0.002	–	NSCLC	64	Anti-PD-1	Retrospective cohort	(29)
	Anti-thyroid antibodies in serum	Anti-Tg antibody, but not anti-TPO associated with thyroid dysfunction	OR_adj_ = 26.5; 95% CI 8.18–85.8; p < 0.001 | p > 0.05	–	Solid tumor	168	Anti-PD-1	Retrospective cohort	(30)
Anti-BP180 IgG	Serum IgG directed against BP180, BP230, and type VII collagen measured by ELISA	Anti-BP180 IgG levels associated with dermal irAE	p = 0.04	–	NSCLC	40	Anti-PD-(L)1	Prospective cohort	(31)
Anti-GNAL and anti-CD74	Identification of autoantibodies using recombinant cDNA expression libraries	Anti-GNAL presence associated with hypophysitis | anti-CD74 presence associated with pneumonitis	OR = 2.66; 95%CI 1.14–7.29; p = 0.02 OR = 1.25; 95%CI 1.03–1.52; p = 0.03	AUC = 1 for both	Solid tumor	20 32	Anti-CTLA-4, anti-PD-1, combination	Nested prospective cohort	(32)
Various autoantibodies	Autoantibodies tested with HuProt array covering >19,000 proteins	Enrichment of autoantibodies directed against targets in (auto)immunity pathways associated with grade ≥3 irAEs Classification model based on baseline antibody levels predicts risk of developing severe irAEs	p < 0.05	- Classification models: Sens≥0.89 Spec≥0.85 for each therapy	Melanoma	75	Anti-CTLA-4, anti-PD-1, combination	Prospective cohort	(33)
*Immunogenetics*									
Single-nucleotide polymorphisms (SNPs)	7 SNPs in the *PDCD1, PTPN11, ZAP70* and *IFNG* genes analyzed using whole blood DNA sequencing	PDCD1 804C>T associated with increased irAE risk in exploration cohort, but not in validation cohort	p = 0.039 | p = 0.828	–	NSCLC	161|161	Anti-PD-1	Prospective cohort	(34)
	166 SNPs in 86 immune or cancer related genes analyzed using sequenom MassArray iPLEX assay	SNPs in *UNG*, *IFNW1*, *IFNL4*, *PD-L1*, and *CTLA-4* associated with grade ≥3 irAEs	p < 0.05	AUC = 0.89 Sens = 0.8 Spec = 0.85	Solid tumor	94	Anti-PD-(L)1	Retrospective cohort	(35)
	Polygenic risk scores for skin autoimmunity computed with whole-genome germline sequencing	Psoriasis-associated polygenic risk score associated with skin irAEs	p < 0.05	–	Bladder cancer	220	Anti-PD-L1	Retrospective analysis of clinical trial data	(36)
	miR-146a rs2910164 CC genotype	rs2910164 CC genotype associated with grade 3-4 irAEs	OR = 6.78; 95%CI 1.87–24.6; p = 0.004	–	Solid tumor	167	Anti-PD-(L)1	Prospective cohort	(37)
Human leukocyte antigen (HLA)	HLA-A*02:01 subtype	No association between HLA-A*0201 status and irAEs	Not reported	–	Melanoma	450	Anti-CTLA-4	Retrospective analysis of pooled clinical trial data	(38)
	HLA loci with common variants associated with autoimmune disease	No association of HLA and risk of any irAE HLA-DRB1*11:01 associated with pruritis and HLA-DQB1*03:01 with colitis	Not reported OR = 4.53; p = 0.002 | OR = 3.94; p = 0.017	–	Melanoma, NSCLC	102	Anti-CTLA-4, anti-PD-1, combination	Prospective cohort	(39)
	HLA subtypes in patients with ICI-induced diabetes	Higher HLA-DR4 frequency compared to general population and spontaneous type I diabetes is associated with ICI-induced diabetes	p < 0.0001 | p = 0.002	–	Solid tumor	23	Anti-CTLA-4, anti-PD-1, combination	Case series	(40)
	HLA haplotypes in patients with ICI-induced adrenal insufficiency	HLA-DR15 associated with pituitary irAEs	p = 0.0014	–	Advanced cancer	11	Anti-PD-1 or anti-CTLA-4	Case-control study	(41)
*Microbiome*									
Bacteroidetes, Firmicutes	Microbiome composition in fecal samples using 16S rRNA and shotgun metagenomic sequencing	Enrichment of Bacteroidetes in patients without colitis. Lack of pathways involved in vitamin B synthesis associated with increased colitis risk	p < 0.05 | p < 0.05	Sens = 70% spec = 83%	Melanoma	34	Anti-CTLA-4	Prospective cohort	(42)
	Microbiome composition in fecal samples using16S rRNA sequencing	Enrichment of Bacteroidetes in patients without colitis. Enrichment of Firmicutes in patients with colitis	p = 0.011 | p = 0.009	–	Melanoma	26	Anti-CTLA-4	Prospective cohort	(23)
*Other*									
Thyroid stimulating hormone (TSH)	TSH, free T3 and free T4 in serum	TSH ≥5 µIU/ml associated with increased risk of thyroid dysfunction	OR_adj_ = 7.36; 95%CI 1.66–32.7; p = 0.01	–	Solid tumor	168	Anti-PD-1	Retrospective cohort	(30)
	Serum TSH	TSH >2.19 µIU/ml associated with increased risk of thyroid dysfunction	OR = 3.46; 95%CI 1.2–9.8	AUC = 0.66 Sens = 53% Spec = 76%	Melanoma	99	Anti-PD-1, combination	Retrospective cohort	(43)
Soluble CTLA-4	sCTLA-4 in serum by ELISA	>200 pg/ml sCTLA-4 associated with irAEs	OR_adj_ = 3.63; 95%CI 1.14–11.5; p = 0.029	–	Melanoma	113	Anti-CTLA-4	Prospective cohort	(44)

n, number of patients included in the reported analysis (irrespective of irAE presence); irAE, immune-related adverse event; OR, odds ratio; OR_adj_, adjusted odds ratio; 95%CI, 95% confidence interval; Sens, sensitivity; Spec, specificity; AUC, area under the receiver operating curve; NSCLC, non-small cell lung cancer; Anti-PD-(L)1, anti-programmed cell death protein (ligand) 1; anti-CTLA-4, anti-cytotoxic T lymphocyte antigen 4; combination, combined anti-CTLA-4 plus anti-PD-1 therapy; ICI, immune checkpoint inhibitor; CD, cluster of differentiation; sCD, soluble CD; Tregs, regulatory T-cells; NLR, neutrophil to lymphocyte ratio; PNI, prognostic nutrition index; PLR, platelet to lymphocyte ratio; IL, interleukin; G-CSF, granulocyte colony-stimulating factor; GM-CSF, granulocyte-macrophage colony-stimulating factor; FGF, fibroblast growth factor; IFN, interferon; CXCL, chemokine C-X-C motif ligand; CCL, chemokine C-C motif; Tg, Thyroglobulin; TPO, Thyroid peroxidase; BP, bullous pemphigoid; IgG, immunoglobulin G; GNAL, guanine nucleotide-binding protein G subunit alpha; SNPs, single-nucleotide polymorphisms; HLA, human leukocyte antigen; TSH, thyroid stimulating hormone; T3, Triiodothyronine; T4, thyroxine; miR, micro-RNA; ELISA, enzyme-linked immunosorbent assay.

**Table 2:** Potential biomarkers for irAE development during ICI treatment.

Potential biomarker	Determinant	Results	Significance	Accuracy	Cancer type	n	Treatment	Study design	References
*Cellular*									
Leukocytes	Peripheral blood cell counts after irAE onset**	Increase in leukocyte count and decrease in relative lymphocyte count associated with grade ≥3 irAEs in univariable, but not multivariable analysis	OR_adj_ = 1.13; 95%CI 0.99–1.29; p = 0.074 | OR_adj_ = 1.18; 95%CI 0.94–1.48; p = 0.15	–	Melanoma	101	Anti-PD-1	Retrospective cohort	(45)
Eosinophils	Leukocyte subsets at 1, 3 and 6 months*	Absolute eosinophil count after 1 month associated with grade ≥2 irAEs	OR_adj_ = 1.002; 95%CI 1.000–1.004; p = 0.027	–	Solid tumor	167	Anti-PD-1, combination	Retrospective cohort	(20)
	Absolute and relative eosinophil counts after 1, 3 and 6 months*	Relative eosinophil count at 1 month >3.2% associated with endocrine irAEs	OR = 5.11; 95%CI 1.23-21.3; p = 0.025	Sens = 67% Spec = 72%	Melanoma	44	Anti-PD-1	Retrospective cohort	(21)
	Relative eosinophil count after onset of dermal irAEs compared with baseline**	Relative eosinophil count increased during treatment in patients with dermal irAEs, but not in those without	p = 0.006 vs. p = 0.19	–	Melanoma	17	Anti-CTLA-4	Nested case control	(46)
Lymphocytes	Leukocyte subsets at 1, 3 and 6 months*	Lymphocyte count >2,000 at 1 month associated with risk of grade ≥2 irAEs, any irAEs and irAEs requiring treatment	OR_adj_ = 1.81; 95%CI 1.03–3.25; p = 0.039 | p < 0.05 | p < 0.01	–	Solid tumor	167	Anti-PD-1, combination	Retrospective cohort	(20)
	T-cell receptor β-chain sequencing in purified T cells from blood at baseline and before irAE occurrence (median 13 days; IQR 2-24)*	Clonal expansion of ≥55 CD8 T-cell clones associated with grade ≥2 irAEs	p < 0.0001	Sens = 100% Spec = 42% AUC = 0.87	Prostate cancer	27	Anti-CTLA-4 + androgen deprivation	Retrospective analysis of clinical trial data	(47)
	T-cell receptor β-chain sequencing in purified T cells from blood at baseline and week 2*	Clonal expansion of more T-cell clones and higher number of newly emerging T-cell clones are associated with irAEs; decline in T-cell clonality in patients with irAEs at week 2 versus baseline	p = 0.028 | p = 0.042; p = 0.023	–	Prostate cancer	21	Anti-CTLA-4 + GM-CSF	Retrospective analysis of clinical trial data	(48)
	Circulating B-cell changes after the first cycle of treatment compared to baseline*	≥30% decline in B cells and doubling of CD21lo cells or plasmablasts associated with grade ≥3 irAEs	p < 0.001	–	Melanoma	23	Combination of anti-CTLA-4 and anti-PD-1	Prospective cohort	(49)
*Cytokines/Chemokines*									
IL-6	Pre- and post-treatment serum samples tested for IL-6, TNF-α, IFN-γ, IL-8 and IL-17A analysis^?^	Increased IL-6 levels in irAE patients post-treatment compared to pre-treatment, not in patients without irAEs	p = 0.018	–	Melanoma	20	Anti-PD-1	Case series	(50)
Various cytokines/chemokines	65 cytokines/chemokines profile at week 1-6 validated in separate cohort*	CYTOX score (G-CSF, GM-CSF, fractalkine, FGF-2, IFNα2, IL1a, IL1B, IL1RA, IL2, IL12p70, and IL13) associated with irAEs that required ICI discontinuation or immunosuppression	p = 0.0168	AUC = 0.70	Melanoma	49	Anti-PD-1 or combination	Prospective cohort	(26)
CXCL9, CXCL10	40 cytokines in serum tested at baseline and after 2-3 and 6 weeks*	A greater increase of CXCL9 and CXCL10 at week 6 in irAE patients compared to non-irAE patients	p < 0.05	–	Solid tumor	42	Anti-PD-(L)1	Prospective cohort	(28)
CCL5	75 serum proteins measured by Milliplex MAP assay at baseline and after 4 weeks*	CCL5 levels at 4 weeks in irAE patients increased compared to non-irAE patients	p < 0.05	–	NSCLC	32	Anti-PD-1	Prospective cohort	(51)
sCD163	Serum levels of sCD163 and CXCL5 measured at day 0 and day 42*	Change from baseline of sCD163 level at day 42 was higher in irAE patients compared to non-irAE patients	p = 0.0018	Sens = 73% Spec = 75%	Melanoma	46	Anti-PD-1	Retrospective cohort	(52)
*Autoantibodies*									
Anti-thyroid antibodies	Anti-Tg and anti-microsomal antibodies measured at baseline and every cycle during treatment*^/^**	Anti-Tg present in 80% of patients with thyroid dysfunction compared to 8% of patients without	p < 0.0001	–	NSCLC	48	Anti-PD-1	Prospective cohort	(53)
	Autoantibodies tested at baseline and after 4 weeks*	Increased levels of anti-Tg and anti-TPO at 4 weeks compared to baseline associated with thyroid irAEs	p = 0.012 | p = 0.048	–	Advanced cancer	26	Anti-PD-1, anti-CTLA-4, or combination	Prospective cohort	(27)
Anti-GNAL and anti-ITM2B antibodies	Pre-treatment and post-treatment before irAE analysis with recombinant cDNA libraries	Stronger increase of anti-GNAL and anti-ITM2B in patients with hypophysitis	p < 0.001 | p < 0.001	AUC = 1 | AUC = 1	Solid tumor	20	Anti-CTLA-4, anti-PD-1, combination	Nested prospective cohort	(32)
*Other*									
CRP	CRP levels measured at baseline and just before or at irAE onset*^/^**	CRP levels rose from mean 8.4 mg/L at baseline to 52.7 mg/L at irAE onset	p < 0.0001	–	Melanoma	37	Anti-CTLA-4, anti-PD-1, combination	Case series	(12)
	CRP levels at baseline, at irAE onset and after tocilizumab administration**	CRP levels increased from median 23 mg/L at baseline to 109 mg/L at onset of irAEs and decreased to 19 mg/L after irAE treatment	p < 0.00001	–	Advanced cancer	34	Anti-PD-1	Case series	(54)
CD177	Gene expression profiling of 9697 non-control probe sets on whole blood samples at baseline, week 3 and 11*	Increased CD177 gene expression at 3 weeks in patients with GI irAEs compared to non-irAE patients and compared to baseline	p = 0.0076	–	Melanoma	162	Anti-CTLA-4	Retrospective analysis of clinical trial data	(55)

*before onset of irAEs, either obtained through collection at regular time points or at last measurement before irAE onset; **at onset of irAEs; ^?^timing not clear from report

n, number of patients included in the reported analysis (irrespective of irAE presence); irAE, immune-related adverse event; OR, odds ratio; OR_adj_, adjusted odds ratio; 95%CI, 95% confidence interval; IQR, interquartile range; Sens, sensitivity; Spec, specificity; AUC, area under the receiver operating curve; NSCLC, non-small cell lung cancer; Anti-PD-(L)1, anti-programmed cell death protein (ligand) 1; anti-CTLA-4, anti-cytotoxic T lymphocyte antigen 4; combination, combined anti-CTLA-4 plus anti-PD-1 therapy; ICI, immune checkpoint inhibitor; GM-CSF, granulocyte-macrophage colony-stimulating factor; CD, cluster of differentiation; sCD, soluble CD; IL, interleukin; TNF, tumor necrosis factor; IFN, interferon; G-CSF, granulocyte colony-stimulating factor; FGF, fibroblast growth factor; CXCL, chemokine C-X-C motif ligand; CCL, chemokine C-C motif; Tg, Thyroglobulin; TPO, Thyroid peroxidase; GNAL, guanine nucleotide-binding protein G subunit alpha; ITM, integral membrane protein; CRP, c-reactive protein.

### Immune Cells

High neutrophil-to-lymphocyte ratio (NLR) was shown to correlate with worse survival in multiple ICI treated malignancies (56), which might suggest that ICI-induced anti-tumor responses are less effective in these patients. In line with this hypothesis, patients with high NLR might also have a lower risk of immune-mediated toxicities. Indeed, among 391 anti-PD-1 treated patients with various types of malignancies, Eun et al. demonstrated that patients with an NLR ≥ 3 less often experienced irAEs than patients with a low NLR (17). However, the median follow-up time in this study was less than 7 weeks and irAEs were reported in only 17% of patients. In their study amongst 102 anti-PD-1 treated melanoma patients, Peng et al. reported that patients with NLR>5 experienced less irAEs (18). Additionally, they observed that a prognostic nutrition index (PNI), which is calculated from the serum albumin level and total lymphocyte count, of 45 or higher was correlated with increased risk of irAEs. In a third study consisting of 173 anti-PD-(L)1 treated NSCLC patients, Pavan et al. reported that patients with an NLR < 3 had an increased risk of irAEs in univariable analysis, as did patients with a platelet-to-lymphocyte ratio (PLR) < 180. In multivariable analysis however, only a low-PLR remained significant, which was confirmed in a competing risk analysis accounting for death (19). Altogether, these three studies support the hypothesis that high NLR may be associated with less irAEs. However, confirmational studies accounting for time at risk should be conducted to dissect the risk of irAEs from survival.

In a retrospective analysis of 167 anti-PD-1 treated patients, Diehl et al. observed that patients with an absolute lymphocyte count (ALC) > 2,000 at baseline and at one month into therapy significantly more often developed grade ≥ 2 irAEs (20). Additionally, a 10% increase of overall leukocyte count and relative lymphocyte count were associated with grade ≥ 3 irAEs in a univariable analysis of 101 anti-PD-1 treated melanoma patients. However, this association was not significant in adjusted analyses (45).

Since a pathogenic role of eosinophils has been proposed in various autoimmune diseases such as inflammatory bowel disease, primary biliary cirrhosis, bullous pemphigoid, and eosinophilic myocarditis (57), eosinophils might be an interesting biomarker of irAEs. Indeed, two studies reported increased peripheral blood eosinophil counts both at baseline and one month after start of anti-CTLA-4 or anti-PD-1 in patients with irAEs compared to those without (20, 21). While Diehl et al. aimed to study the relationship between lymphocyte counts and irAEs in 167 solid tumor patients treated with anti-PD-1 or anti-PD-1/CTLA-4 combination therapy, they also observed that absolute eosinophil counts correlated to ≥2 grade irAEs. However, the OR was just 1.34 for increments of 100, which may not be clinically relevant. In an analysis of 44 anti-PD-1 treated melanoma patients, Nakamura et al. showed that elevated baseline absolute eosinophil levels (>240/µl) and a relative eosinophil count after one month >3.2% correlated to endocrine irAEs specifically. A third study by Jaber et al. in nine anti-CTLA-4 treated melanoma patients showed increased blood eosinophil levels after onset of dermal irAEs compared to pre-treatment levels, which was not found in 8 patients without irAEs (46). In contrast to these studies, Eun et al. found no correlation of eosinophil counts with toxicity in 391 anti-PD-1 treated patients with various types of malignancies (17). Still, these results demonstrate that eosinophils might play a role in the development of irAEs and could pose as interesting biomarkers.

As key players in cancer immunology and main target cells of ICI therapy (58), T cells are of particular interest in the quest for biomarkers of irAEs. Only few studies reported on specific T-cell subsets. To analyze myeloid-derived suppressor cell (MDSC) and T-cell subsets, Damazzu et al. conducted multi-color flow cytometry on fresh whole blood samples of 44 anti-CTLA-4 treated melanoma patients at baseline and at 12 week time intervals after treatment initiation (22). Although they found no difference in CD3^+^ count or CD3^+^/CD4^+^ ratio, they demonstrated lower percentages of PD-1-expressing CD4^+^ and CD8^+^ T cells at baseline in patients with grade ≥3 irAEs compared to those without. In addition, significant upregulation of PD-1 expression was observed on CD4^+^ T cells in patients without irAEs. In patients with irAEs, PD-1 was also upregulated on CD4^+^ T cells but this did not reach statistical significance. In CD8^+^ T cells, PD-1 expression was significantly upregulated in both patients with irAEs and those without. There were no differences in MDSC subsets in patients with or without severe toxicities. Chaput et al. reported that patients with anti-CTLA-4-induced colitis had higher absolute CD4^+^ T-cell numbers at baseline in peripheral blood compared to non-colitis patients according to flow-cytometry analysis. The difference did not reach statistical significance (p = 0.053) (23). Whereas Damazzu et al. did not report on regulatory T-cells (Tregs), Chaput et al. reported that the percentage of Tregs at baseline in patients with anti-CTLA-4-induced colitis was significantly lower compared to non-colitis patients, although absolute numbers were not significantly different. Taken together, although correlations of some T-lymphocyte subsets with irAEs have been reported, further research is needed to prove their predictive value.

Changes in T-cell receptor repertoire in peripheral blood early during treatment were linked to irAEs in two studies reporting on anti-CTLA-4 treatment combined with either androgen deprivation therapy (ADP) or granulocyte-macrophage colony-stimulating factor (GM-CSF) (47, 48). Both studies used next-generation sequencing of CDR3 regions in rearranged T-cell receptor (TCR) β-chains and reported that a more diverse T-cell repertoire is observed in patients with irAEs. Subudhi et al. demonstrated in 16 anti-CTLA-4 + ADP treated patients, that the number of expended clonotypes of CD8^+^ (but not CD4^+^) T cells just before toxicity compared to baseline was significantly higher in 11 patients with grade ≥2 irAEs compared to five patients without. An expansion of ≥55 T-cell clones was reported to be 100% sensitive and 42% specific of grade ≥2 irAEs, which was confirmed in a validation cohort of 11 patients. Similarly, Oh et al. reported that an increase in frequency of preexisting clonotypes in the blood was associated with irAEs in a study with 35 anti-CTLA-4 + GM-CSF treated patients. In addition, irAE occurrence was also associated with an increase of newly detected TCR clones. Overall clonality declined in CD4^+^ and CD8^+^ T cells at week 2 after treatment initiation in all patients, which was significant in patients with irAEs but not in patients without. Taken together, these data show that anti-CTLA-4-induced increase in T-cell diversity preceded irAEs. This observation is not surprising considering that CTLA-4 suppresses T cells early after activation, thereby preventing T-cell reactions against self-antigens. Thus, blocking CTLA-4 might allow activation of previously suppressed self-reactive T-cell clones and the degree of activation expressed as early diversification of T-cell repertoire after treatment might be associated with occurrence of irAEs. As PD-1 acts later after T-cell activation in the peripheral tissue, the results obtained by Subudhi and Oh et al. may not be translatable to anti-PD-1 therapies.

Three recent papers simultaneously identified B cells as major actors in ICI therapy by demonstrating that B-cell tumor infiltration and formation of tertiary lymphoid tissues was correlated with better prognosis (59–61). The role of B cells and their predictive value in irAEs has however been less well studied. Only one study on B-cell changes has been reported. By using flow cytometry on PBMCs of 23 anti-CTLA-4 + anti-PD-1 treated melanoma patients, at baseline and during follow-up, Das et al. demonstrated that an early decline in B-cell numbers of more than 30% together with a doubling of CD21^lo^ B cells or plasmablasts preceded grade ≥3 irAEs (49). The severity of the decline in B cells was directly correlated with the time of onset of the irAEs.

### Cytokines/Chemokines

Various cytokines, such as interleukin (IL)-6 and IL-17, are associated with inflammation and autoimmune diseases (62) and have therefore been proposed as predictors of irAEs.

IL-6, produced by almost all stromal and immune cells, has broad context-dependent pro-inflammatory effects on innate and adaptive immunity and is also important in epithelial homeostasis (62, 63). Two studies reported that lower baseline serum IL-6 was associated with irAEs in anti-CTLA-4 treated melanoma patients. In blood of 140 anti-CTLA-4 treated melanoma patients at baseline, Valpione et al. analyzed LDH, S-100, CRP, β-2-microglobulin, VEGF, IL-2, and IL-6 levels using various techniques including immune-enzymatic methods as well as lymphocyte subsets using flow cytometry (24). They observed that only low baseline IL-6 serum levels (<2.5ng/L) and female sex were correlated with increased risk of grade ≥3 irAEs after adjustment for follow-up time. Similarly, Chaput et al. observed lower levels of IL-6, IL-8, and sCD25 in patients with anti-CTLA-4-induced colitis using multiplex assays (23). Furthermore, Tanaka et al. reported that serum IL-6 levels increased during treatment in all six patients with anti-PD-1-induced psoriasis and all seven patients with other irAEs, while IL-6 levels declined in five out of seven control patients without irAEs according to a multiplex assay (50). In agreement with these findings, C-reactive protein (CRP), a general marker of inflammation, which is sometimes suggested as surrogate marker of IL-6 (64), was reported to rise in patients with irAEs just preceding or at onset of irAEs in two studies (presented under “other” section in **Table 2**). In 37 ICI-treated melanoma patients with 88 cases of irAEs, Abolhassani et al. observed a statistically significant increase of the mean CRP level from 8.4 mg/L at baseline to 52.7 mg/L just before or at onset of irAEs for all 88 cases (12). Similarly, Stroud et al. reported a statistically significant increase in CRP from a median of 23 mg/L at baseline to 109 mg/L at time of irAEs, which decreased to 19 mg/L after tocilizumab (anti-IL-6) in 87 anti-PD-1 treated patients (54). However, a remarkably high percentage of these patients (39%) required tocilizumab to treat the irAE, which suggests selection of patients. Furthermore, neither Abolhassani, nor Stroud et al. reported data on CRP alterations in patients without toxicity, so a rise in CRP during ICI treatment irrespective of irAE development could not be ruled out.

Five additional studies reported on the correlation between numerous cytokines/chemokines and irAEs using multiplex assays consisting of 18 to 65 variables in cohorts ranging from 26 to 49 patients, which yielded contradicting results (**Supplementary Table S1**). In contrast to the studies discussed above, none of these five studies reported a correlation between IL-6 and irAEs (25–28, 51). Nevertheless, IL-6 and CRP could be promising predictors of irAEs if validated in sufficiently powered cohorts.

In a study of 35 anti-CTLA-4 treated patients, Tarhini et al. observed higher baseline IL-17 levels to be associated with grade ≥3 irAEs and colitis, using a multiplex panel of 36 cytokines and chemokines (25). IL-17 is a pro-inflammatory cytokine mainly produced by T-helper 17 (Th17) cells, and contributes to the pathogenesis of several autoimmune diseases, such as psoriasis and rheumatoid arthritis (65). IL-17 seems to have both oncogenic and anti-tumor effects, as comprehensively reviewed by Qian et al. *(* 66 *)* and Th17 cells have been proposed as important actors in irAEs (67). In line with this, colonic mRNA expression of IL-17A was shown to be upregulated in nine anti-CTLA-4-induced colitis patients compared to eight healthy controls as well as interferon-γ, FoxP3, IL-10 and TNF-like molecule TL1A in a study of mRNA expression of 14 inflammatory mediators in colon tissue biopsies (68). In the studies on anti-PD-(L)1 or combination therapy treated patients however, no significant correlation of IL-17 with toxicity was observed.

In an analysis of 65 cytokines or chemokines in combination ICI-treated patients, an aggregated CYTOX score consisting of 12 cytokines/chemokines was associated with severe irAEs in exploratory (n = 58) and validation (n = 49) cohorts at baseline and 1-6 weeks after treatment initiation (26). Three of these 12 parameters (IL-1β, IL-2, and GM-CSF) were also observed to be associated with irAEs among a multiplex assay of 18 cytokines/chemokines in 26 anti-PD-1, anti-CTLA-4 or combined therapy-treated patients (27). Additionally, this study found an early decrease of IL-8, G-CSF, and MCP-1 during treatment to be associated with thyroid irAEs.

With their role in the migration of immune cells into tissues, chemo-attractants may play a crucial role in irAE development. In a 40-plex assay in 65 patients receiving ICIs, Khan et al. observed that CXCL9 (monokine induced by gamma interferon [MIG]), CXCL10 (IFN-γ induced protein -10 [IP-10]), CXCL11 (interferon-inducible T cell α chemoattractant [I-TAC]), and CCL19 (macrophage inflammatory protein 3β [MIP-3β]) were lower at baseline in patients with irAEs compared to those without (28). Furthermore, they reported a greater increase of CXCL9 and -10 in patients with irAEs at 2-3 and 6 weeks after treatment initiation. Interaction of the chemokines CXCL9, -10, and -11 with their receptor CXCR3 can elicit differentiation of naïve T cells into Th1 cells and plays a role in the recruitment of these Th1 cells, cytotoxic T cells and natural killer (NK) cells, as comprehensively reviewed by Tokunaga et al. (69). Another chemoattractant, RANTES (regulated on activation, normal T cell expressed and secreted/CCL5) was observed to be higher in 11 anti-PD-1 treated patients with irAEs compared to the 21 patients without 4 weeks after treatment initiation, but not at baseline (51).

Fujimura et al. reported a greater increase of soluble CD163 after 42 days of anti-PD-1 treatment in 22 patients with irAEs compared to the 24 without, using enzyme-linked immunoassays (ELISA) on serum (52). However, this seems to be mainly due to 3 patients with major increases, as sCD163 levels actually decreased in half of the patients with irAEs. In fact, the authors report a receiver operating characteristic (ROC) curve in which the cut-off level of 21.3% is based on both increase or decrease of sCD163 serum levels.

### Autoantibodies

The observation that pre-existing autoantibodies are present in patients with several specific types of irAEs has led to the hypothesis that these play a role in modulation of irAE pathogenesis (4). For example, increased anti-thyroid antibody levels at baseline or during anti-PD-1 treatment were associated with thyroid dysfunction in three studies (29, 30, 53). The study of Maekura et al. included 64 anti-PD-1 treated NSCLC patients of which serum thyroid peroxidase and thyroglobulin antibody levels were determined with an electrochemiluminescence immunoassay. Five patients developed hyperthyroidism, and this was significantly and positively correlated to the presence of thyroid peroxidase and thyroglobulin antibodies at baseline. A second study by Kimbara et al. in 168 anti-PD-1 treated solid tumor patients reported baseline thyroglobulin antibody levels to be significantly correlated to later thyroid dysfunction after multivariate analysis. However, no correlation of thyroid peroxidase antibodies with thyroid dysfunction was observed in this study. Anti-thyroid antibodies determined during treatment have also been shown to correlate to thyroid dysfunction occurrence. Osorio et al. studied 48 anti-PD-1 treated NSCLC patients and found that 80% of patients with thyroid dysfunction (8 out of 10 patients) displayed anti-thyroglobulin and/or anti-microsomal antibodies compared to 7.8% of patients who did not develop thyroid dysfunction (3 out of 38 patients). Not all patients with anti-thyroglobulin antibodies developed thyroid dysfunction during the study. The majority of patients, 7 out of 11, who were positive for anti-thyroglobulin antibodies developed these antibodies during anti-PD-1 treatment. For six out of these seven patients, antibody presence coincided with thyroid dysfunction onset. Furthermore, Kurimoto et al. studied thyroglobulin and thyroid peroxidase autoantibodies in 26 advanced malignancy patients. Autoantibody levels were determined using an electrochemiluminescence immunoassay. They observed that levels of both autoantibodies showed a more significant increase after four weeks of treatment in patients developing irAEs compared to patients who did not develop irAEs (27). Interestingly, these four studies identified predictive value of anti-thyroid antibodies for irAE development at various time points. Based on the presented data, the strongest predictive value for this biomarker seems to be at baseline.

Various other autoantibodies have been associated with irAE development. One study tested anti-BP-180 IgG (which is associated with bullous pemphigoid) levels by means of ELISA. The authors demonstrated that elevated baseline levels of these antibodies were associated with skin irAEs, but not with irAEs in general in 40 anti-PD-(L)1 treated patients included in the study (31). Using ELISA, baseline anti-GNAL and elevation of both anti-GNAL and anti-ITM2B antibodies during treatment were shown to be associated with hypophysitis in 20 patients and anti-CD74 antibodies with pneumonitis occurrence in 32 patients with solid tumors (32). Finally, one study used a human proteome array covering >19,000 human proteins to assess the presence of autoantibodies against these proteins in serum samples of 75 ICI-treated melanoma patients (33). This study demonstrated a differential expression of autoantibodies in patients with grade ≥3 irAEs compared to those with no or mild irAEs for 914 autoantibodies in 37 anti-CTLA-4 treated patients, for 723 autoantibodies in 27 anti-PD-1 treated patients, and for 1161 autoantibodies in 11 combined anti-CTLA-4 plus anti-PD-1 treated patients. Interestingly, there was only minor overlap in differential expression of autoantibodies in anti-CTLA-4 versus combined and anti-PD-1 versus combined therapy (99 and 54 autoantibodies respectively). Pathway analysis on the protein antigen targets revealed involvement of proteins associated with (auto)immunity. A support vector machine (SVM) classification model was developed to classify patients according to their risk of developing severe immunotherapy-related toxicity based on specific antibody levels in baseline sera. SVM model testing using “curated” antibody lists with lower numbers of antibodies (n = 45 for anti-CTLA-4, n = 25 for anti-PD-1, n = 575 for combination treatment) revealed a high sensitivity (>0.89) and specificity (>0.85) for all treatment groups.

Although autoantibodies have been found that correlate with irAE development, the results certainly warrant further investigation. Remarkably, the strongest correlation of specific autoantibodies with irAE development was seen at baseline. It is well known that organ-specific autoantibodies can be present before onset of clinical symptoms of autoimmune diseases (70). Therefore, patients who present with autoantibodies at baseline might already be prone to develop autoimmune disease irrespective of ICI treatment. Still, ICIs might accelerate the development of immunological disease in these patients. The study of Gowen et al. suggests that prediction models based on baseline random antibody signatures could be further developed as biomarkers to predict toxicity from immunotherapy.

### Immunogenetics

Several single-nucleotide polymorphisms (SNPs) in immune-associated gene loci and human leukocyte antigen (HLA) profiles have been described to be associated with irAEs. Associations with anti-PD-(L)1 related irAEs were found for SNPs in UNG, IFNW1, IFNL4, PDCD1, PD-L-1 and CTLA-4, although this could not be confirmed in a validation cohort (34, 35). Both these studies used a different approach to find useful SNPs. The study of Bins et al. composed an exploration cohort and a validation cohort, both consisting of 161 NSCLC patients. They investigated seven specific SNPs in the PDCD1, PTPN11, ZAP70, and IFNG genes by means of DME Taqman allelic discrimination assays. Specifically, the 804C>T SNP in the PDCD1 gene was shown to correlate to increased risk of irAE development. The other study by Refae et al. selected genes that have been reported in literature to be of relevance for the immune and/or cancer response, which resulted in 166 SNPs in 86 genes. These were analyzed in 94 solid tumor patients with a sequenom MassArray iPLEX assay. This resulted in association of SNPs in UNG, IFNW1, IFNL4, PD-L1, and CTLA-4 with grade ≥3 irAE development. In addition to these two studies, Khan et al. recently published a retrospective analysis of clinical trial data of 220 bladder cancer patients (36). By means of whole genome sequencing they calculated polygenic risk scores for skin autoimmunity and found that psoriasis-associated polygenic risk scores were correlated to dermal irAE development (p < 0.05).

A recent study by Marschner et al. demonstrated microRNA-146a to be of importance for irAE development (37). Mouse models deficient for microRNA-146 showed a higher incidence of severe irAEs compared to wild type mice. In addition, severity of irAEs in mice could be lowered by administering a microRNA-146a mimic *in vivo*. Based on this finding, SNP analysis was performed to identify the SNP rs2910164 (C>G), which is known to decrease microRNA-146a expression, in 167 patients with solid tumors, who had been treated with anti–PD-1 or anti–PD-L1 antibodies. Patients with an rs2910164 CC genotype and therefore decreased microRNA-146a expression had a significantly higher risk of grade ≥3 irAEs development compared to patients with a GC or GG genotype. Patients with rs2910164 CC genotype also showed progression-free survival and increased neutrophil counts both at baseline and during ICI therapy. The frequency of the rs2910164 CC genotype in the study population was low (7%), which was comparable to previously published data in the European population (5%–6%), whereas in Asians, the frequency might be higher (33%) (71). Although the rs2910164 CC genotype was significantly enriched in patients with grade 3–4 irAEs (17.9% versus 3.1% in grade 0–2 irAEs), the majority of patients developed severe irAEs without this genotype. Further studies have to be performed to evaluate the use of this SNP as a biomarker.

HLA profiles have been linked to several autoimmune diseases, such as ankylosing spondylitis (72). Four independent studies reported on HLA profile and irAE development (38–41). In a retrospective analysis of pooled clinical trial data of 450 anti-CTLA-4 treated melanoma patients, Wolchok et al. did not observe a significant association between HLA-A*02:01 profile and irAE occurrence (38). However, this study did notice a trend toward increased irAE frequency in HLA-A*02:01-positive patients, although this was only observed in the subgroup of patients treated with 3 mg/kg anti-CTLA-4. The second study looked at HLA haplotyping by means of next generation sequencing in a prospective cohort of 102 metastatic cancer patients. HLA profiles did not correlate to all developed irAEs, but specifically HLA-DRB1*11:01 was shown to be associated with ICI-induced pruritis and HLA-DQB1*01:01 was associated with colitis (39). Yano et al. performed a case-control study on 11 advanced cancer patients who developed ICI-induced adrenal insufficiency. HLA haplotyping showed a positive association between HLA-DR15 and pituitary irAE development compared to a healthy control group. The study of Stamatouli et al. used a reverse sequence-specific oligonucleotide HLA typing method in 23 patients with solid tumors who developed ICI-induced diabetes. They reported the presence of HLA-DR4 in 76% of these patients, which was significantly higher than in the general population (17%) and in a population with type-1 diabetes (42%). It is known that HLA-DR4 is associated with a higher risk to develop diabetes type 1 (73). It is therefore not surprising that patients expressing this HLA type have a higher risk to develop type 1 diabetes after ICI treatment. Taken together, the data show that certain HLA types might be predictive for a specific irAE type. Since HLA types are associated with different autoimmune phenomena and hence different irAEs, a single HLA type might not serve as a good predictive biomarker for development of irAEs.

### Microbiome

It has been well accepted that the microbiome impacts immune homeostasis (74), and microbiome composition has been demonstrated to impact ICI-efficacy (75–77). Two small studies analyzed microbiome composition in relation to irAEs. Dubin et al. prospectively analyzed the baseline microbiome composition of 34 anti-CTLA-4 treated patients of whom 10 developed colitis using 16S rRNA and metagenomic shotgun sequencing. They reported significantly increased fecal abundance of Bacteroidetes phylum at baseline in patients without colitis (42). Furthermore, they found a relative lack of pathways involved in vitamin B synthesis in the patients who developed colitis. Looking at the predictive accuracy of a four-module analysis identified by machine learning, a 70% sensivity and 83% specificity was reported. In another prospective analysis of 26 anti-CTLA-4 treated patients using 16S rRNA sequencing, a significantly lower abundance of Bacteroidetes but higher abundance of Firmicutes at baseline was reported in the nine patients who developed ICI-associated colitis (23). Although the numbers of patients with irAEs in these studies were small, the identification of Bacteroidetes in both studies is remarkable and deserves further validation in larger cohorts.

### Other

Two studies have reported that baseline thyroid stimulating hormone (TSH) was associated with thyroid dysfunction due to anti-PD-1 or combined therapy, although cut-off values used were different (30, 43). Among 168 anti-PD-1 treated patients, Kimbara et al. observed that a baseline TSH level ≥5 µIU/ml was significantly associated with thyroid dysfunction in a multivariable model. Similarly, in 99 melanoma patients treated with either anti-PD-1 monotherapy or combined with anti-CTLA-4, Pollack et al. reported higher TSH levels at baseline to be associated with thyroid dysfunction, although they used a cut-off of 2.19 µIU/ml. Together and in line with the autoantibody data, this suggests that some patients might have had subclinical thyroid disease before the start of ICIs.

Soluble CTLA-4 has been reported to be associated with irAE development at baseline (44). For this study, serum samples of 113 melanoma patients were collected at baseline and tested with a soluble CTLA-4 specific ELISA. They demonstrated that high baseline levels of soluble CTLA-4 of >200 pg/ml resulted in more than three-fold increased risk of any irAE occurrence.

Retrospective analysis of clinical trial data by Shahabi et al. in 162 melanoma patients identified CD177 mRNA expression as a potential biomarker (55). Gene expression profiling was performed on whole blood samples at baseline and three and six weeks after ICI treatment. They reported increased CD177 mRNA expression levels after three weeks of treatment to be correlated with gastro-intestinal irAEs, when compared to baseline and non-irAE patients. CD177 is a neutrophil marker, which is upregulated during inflammatory responses with neutrophil activation (55). However, only a minority of patients developing grade ≥2 gastro-intestinal irAEs showed an elevated mRNA expression of CD177. Therefore, CD177 has a low sensitivity for prediction of gastro-intestinal irAE development that hampers its use as a biomarker.

## Discussion

This review presents an overview of suggested biomarkers in patients undergoing ICI therapy. We conclude that thus far, none of the proposed biomarkers has shown sufficient accuracy in predicting or signaling irAEs to be of value for clinical practice.

Despite the substantial number of studies on irAE biomarkers, some methodological issues in these studies thus far limit their translation to clinical practice. Generally, the number of patients at risk is low. With the minority of patients developing severe irAEs, this results in low statistical power. As a consequence, most studies focused on irAEs of any grade, including low-grade irAEs, which are less reliably graded. More importantly, the clinical significance of these biomarkers is limited, because low-grade irAEs have fewer clinical implications. Moreover, most analyses were performed retrospectively, and some data are based on case series with their inherent risk of selection bias. Measures indicating their predictive or diagnostic accuracy (such as sensitivity and specificity, ROC analysis) were often not reported. Furthermore, many studies regarding on-treatment irAE biomarkers even lack the comparison with non-irAE patients, making it impossible to analyze their accuracy. Lastly, validation cohorts are lacking in most studies. Future prospectively designed studies should be well powered and address these issues, in order to advance the field.

In our search, we focused on studies reporting potential blood-based, microbiome and immunogenetic biomarkers, but did not include the more pathogenesis focused studies reporting histopathological changes in irAE affected tissue, which could evolve into diagnostic biomarkers in the future. The fact that we only searched PubMed could be seen as a limitation. Although we did not search on other databases such as Embase and Cochrane, we do not expect to have missed articles, because we assume that these articles would have been published in journals which are included in the Medline database.

Reviewing the data, it is unlikely that one single biomarker will be specific or sensitive enough to predict irAE development accurately. Given the fact that there are several mechanisms involved in this process (4) and considering the difference in immunological set-up between patients, it is likely that a combination of multiple biomarkers is needed. Pre-treatment biomarkers should focus on risk stratification and the type of treatment most suited to prevent occurrence of irAEs. A combination of genetic markers (either DNA or RNA based), characteristics of the microbiome and pre-clinical signs of autoimmune disease like presence of autoantibodies or expression of cytokines could be of added value herein. During treatment, biomarkers are needed that distinguish between potential irAE, infection or tumor progression and ideally already indicate development of irAE before onset of clinical symptoms. In this setting, a combination of markers that show a high dynamic range like RNA or protein expression together with traditional inflammatory parameters, like CRP or blood cell counts, seems more suited.

Moreover, as mentioned, biomarkers for anti-PD-(L)1 related irAEs will probably differ from anti-CTLA-4 irAE biomarkers, requesting sufficiently powered studies in these separate populations. In analogy with the work of Bigot et al. who developed a score predicting overall survival for patients receiving ICIs in phase 1 trials (78), a risk score for irAE development could be established. Naturally, such a prediction model should be developed and subsequently validated in separate patient cohorts with sufficient power. The complexity of data generated with different methods, i.e., genomics, transcriptomics, proteomics, and microbiome analysis clearly requires new methods of data analysis. Machine learning techniques could be of added value in this setting as have been applied to predict cutaneous adverse events in patients receiving anti–PD-1 immunotherapy (79).

The development of irAEs has been associated with improved response to ICI treatment, as reviewed by Das & Johnson (80). In line with this, some of the irAE biomarkers in this review have also shown associations with ICI responses (18–22, 31, 37, 44). This is not surprising, in view of the common underlying mechanism of irAEs and anti-tumor responses (48). It is important to consider that the patients at increased risk of irAEs could also be the patients deriving the most benefit from ICIs and that aggressive or early irAE management could compromise ICI efficacy (81).

In conclusion, no single blood-based biomarker has been identified to date that has the potential to accurately predict risk of irAE development in patients undergoing ICI treatment. Future prospective studies using standardized sampling and analyses should be performed in well-powered cohorts and focus on combinations of potential biomarkers that are validated in separate cohorts. The overview of suggested biomarkers in this review could be a starting point for further research in order to develop a successful prediction method.

## Author Contributions

JH and DH designed the study. JH and RV performed the systematic literature search and selected studies to be included. DH and KS advised on search and selection strategy. JH and RV drafted the manuscript. DH and KS edited the manuscript. All authors contributed to the article and approved the submitted version.

## Conflict of Interest

KS reports advisory relationships with Novartis, Pierre Fabre, MSD, Abbvie and Bristol Myers Squibb and received honoraria from Novartis and Roche; all outside the submitted work and paid to institution.

The remaining authors declare that the research was conducted in the absence of any commercial or financial relationships that could be construed as a potential conflict of interest.

## Appendix 1: Search strategy

A search was conducted using PubMed with search terms for immune checkpoint inhibitor therapy, immune-related adverse events and biomarker (full search question below). The search on PubMed yielded 3580 results on the 1st of July 2020, of which 35 were included in this review. Five other articles were obtained by means of citation and by using the similar article function on PubMed.

((toxicit*[Title/Abstract] OR (adverse[Title/Abstract] AND (event [Title/Abstract] OR events[Title/Abstract])) OR irAE[Title/Abstract])

AND

(PD-1[Title/Abstract] OR PD1[Title/Abstract] OR anti-PD1[Title/Abstract] OR anti-PD 1[Title/Abstract] OR pembrolizumab[Title/Abstract] OR nivolumab[Title/Abstract]

OR

PD-L1[Title/Abstract] OR anti-PD-L1[Title/Abstract] OR anti-PDL1[Title/Abstract] OR durvalumab[Title/Abstract] OR avelumab[Title/Abstract] OR atezolizumab[Title/Abstract]

OR

CTLA-4[Title/Abstract] OR CTLA4[Title/Abstract] OR ipilimumab[Title/Abstract] OR tremelimumab[Title/Abstract] OR cemiplimab[Title/Abstract] OR anti-CTLA-4[Title/Abstract] OR anti-CTLA4[Title/Abstract] OR

Anti cytotoxic T-lymphocyte-associated protein 4[Title/Abstract] OR anti-CD152[Title/Abstract]

OR

checkpoint inhibit*[Title/Abstract] OR immune checkpoint[Title/Abstract])

AND

(predict*[Title/Abstract] OR associate*[Title/Abstract] OR biomarker*[Title/Abstract] OR signal*[Title/Abstract] OR increase*[Title/Abstract] OR decrease*[Title/Abstract]))
