# Supplementary material for: Biomarkers of Checkpoint Inhibitor Induced Immune-Related Adverse Events—A Comprehensive Review
Source: Front Oncol. 2021 Feb 11;10:585311. doi: 10.3389/fonc.2020.585311 (PMC7905347; doi:10.3389/fonc.2020.585311)
Supplement: Supplementary file 1 [file Table_1.pdf]

Supplementary data

Biomarkers of checkpoint inhibitor induced immune-related adverse events – a comprehensive review

Josefien W. Hommes, Rik J. Verheijden, Karijn P.M. Suijkerbuijk, Dörte Hamann

Frontiers in Oncology 2020

Supplementary table 1: Summary of cytokines measured per study and their statistically significant associations with immune-related adverse events (irAEs)

	Baseline							EDT*	ΔirAE-baseline**			
	Lim et al. 2019 (41)	S. Khan et al. 2019 (42)	Oyanagi et al. 2019 (43)	Tarhini et al. 2015 (44)	Kurimoto et al. 2020 (45)	Valpione et al. 2018 (37)	Chaput et al. 2017 (28)	Lim at al. 2019 (41)	S. Khan et al. 2019 (42)	Oyanagi et al. 2019 (43)	Tanaka et al. 2017 (38)	Fujimura et al. 2018 (51)
Number of patients	49	42	32	35	26	140	18	49	42	32	20	46
6Ckine/ CCL21												
Angiopoietin-2												
BCA-1/ CXCL13												
BMP-9												
CRP												
CTACK/ CCL27												
EGF												
ENA-78/ CXCL5												
Endoglin												
Endothelin-1												
Eotaxin/ CCL11												
Eotaxin-2/ CCL24												
Eotaxin-3/ CCL26												
FGF-basic												
FGF-1												
FGF-2												
Flt-3L												
Follistatin												
Fractalkine/ CX3CL1												
G-CSF												
GM-CSF					‡							
GCP-2/ CXCL6												
GRO-basic												
GRO-α/ CXCL1												
GRO-β/ CXCL2												
HB-EGF												
HGF												
I-309/ CCL1												
IFN-α												
IFN-γ												
IL-1α												
IL-1β					‡							
IL-1RA												
IL-2					‡							
IL-2RA/ sCD25							†					
IL-3												
IL-4												

	Baseline							EDT*	ΔirAE-baseline**			
	Lim et al. 2019 (41)	S. Khan et al. 2019 (42)	Oyanagi et al. 2019 (43)	Tarhini et al. 2015 (44)	Kurimoto et al. 2020 (45)	Valpione et al. 2018 (37)	Chaput et al. 2017 (28)	Lim at al. 2019 (41)	S. Khan et al. 2019 (42)	Oyanagi et al. 2019 (43)	Tanaka et al. 2017 (38)	Fujimura et al. 2018 (51)
IL-5												
IL-6							†				¶	
IL-7												
IL-8/ CXCL8							†					
IL9												
IL-10												
IL12P40												
IL12P70												
IL-13												
IL-15												
IL-16												
IL-17				†								
IL-18												
IL-20												
IL-21												
IL-23												
IL-28A/ IFNL2												
IL-33												
IP-10/ CXCL10												
I-TAC/ CXCL11												
Leptin												
LIF												
MCP-1/ CCL2												
MCP-2/ CCL8												
MCP-3/ CCL7												
MCP-4/ CCL13												
MDC/ CCL22												
MIF												
MIG/ CXCL9												
MIP-1α/ CCL3												
MIP-1β/ CCL4												
MIP-1d/ CCL15												
MIP-3α/ CCL20												
MIP-3β/ CCL19												
MPIF-1/ CCL23												
PDGF-AA												
PDGF-AB/BB												
PGE2												
PLGF												
RANTES/ CCL5												

	Baseline								EDT*	ΔirAE-baseline**			
	Lim et al. 2019 (41)	S. Khan et al. 2019 (42)	Oyanagi et al. 2019 (43)	Tarhini et al. 2015 (44)	Kurimoto et al. 2020 (45)	Valpione et al. 2018 (37)	Chaput et al. 2017 (28)		Lim at al. 2019 (41)	S. Khan et al. 2019 (42)	Oyanagi et al. 2019 (43)	Tanaka et al. 2017 (38)	Fujimura et al. 2018 (51)
sCD40L													
sCD163													
SCF													
SCYB16/ CXCL16													
SDF-1/ CXCL12													
TARC/ CCL17													
TGF-α													
TGF-β1													
TGF-β2													
TGF-β3													
TNF-α													
TNF-β/LT-α													
TPO													
TRAIL													
TSLP													
VEGF													
VEGF-A													
VEGF-C													
VEGF-D													

*EDT= early during treatment=1-6 weeks after start of therapy

** ΔirAE-baseline= increase/decrease at onset of irAE compared to baseline

† associated with colitis particularly

‡ associated with thyroiditis particularly

¶ associated with psoriasis particularly

Legend

Grey = parameter was analysed but not significantly correlated with irAE

Green = higher/increased in patients with irAE

Orange = lower/decreased in patients with irAE

Yellow = prediction model of multiple parameters
